# Genome Sequences of Two GH Clade SARS-CoV-2 Strains Isolated from Patients with COVID-19 in South Korea

**DOI:** 10.1128/MRA.01384-20

**Published:** 2021-01-07

**Authors:** Minwoo Kim, Youn-Jung Lee, Jae Sun Yoon, Jin Young Ahn, Jung Ho Kim, Jun Yong Choi, Jong-Won Oh

**Affiliations:** a Department of Biotechnology, Yonsei University, Seoul, South Korea; b Division of Infectious Diseases, Department of Internal Medicine, Yonsei University College of Medicine, Seoul, South Korea; DOE Joint Genome Institute

## Abstract

We report the genome sequences of two GH clade severe acute respiratory syndrome coronavirus 2 (SARS-CoV-2) strains isolated from nasopharyngeal swabs from patients with coronavirus disease 2019 (COVID-19) in South Korea. These strains had two mutations in the untranslated regions and seven nonsynonymous substitutions in open reading frames, compared with Wuhan/Hu-1/2019, showing 99.96% sequence identity.

## ANNOUNCEMENT

A novel coronavirus, severe acute respiratory syndrome coronavirus 2 (SARS-CoV-2), belonging to the genus *Betacoronavirus* of the family *Coronaviridae*, emerged in December 2019 in Wuhan, China (1, 2). The ongoing pandemic of coronavirus disease 2019 (COVID-19) caused by SARS-CoV-2 poses an unprecedented threat to global health and health care systems, with over 69 million laboratory-confirmed COVID-19 cases and more than 1.5 million casualties as of December 2020 (3).

Here, we report the genome sequences of two SARS-CoV-2 strains isolated from two patients with COVID-19 who were hospitalized in Severance Hospital, Yonsei University (Seoul, South Korea), in June 2020 during the COVID-19 pandemic. Nasopharyngeal swab specimens were collected on the day on which the patients tested positive for COVID-19 by real-time quantitative PCR using the Allplex 2019-nCoV assay kit (Seegene, Seoul, South Korea), with critical threshold values of 13.92 for patient 6 and 14.34 for patient 8. All of the studies were approved by the institutional review board (IRB) of Severance Hospital, Yonsei University Healthcare System, with written informed consent from the patients (IRB protocol number 4-2020-0076).

**FIG 1:**
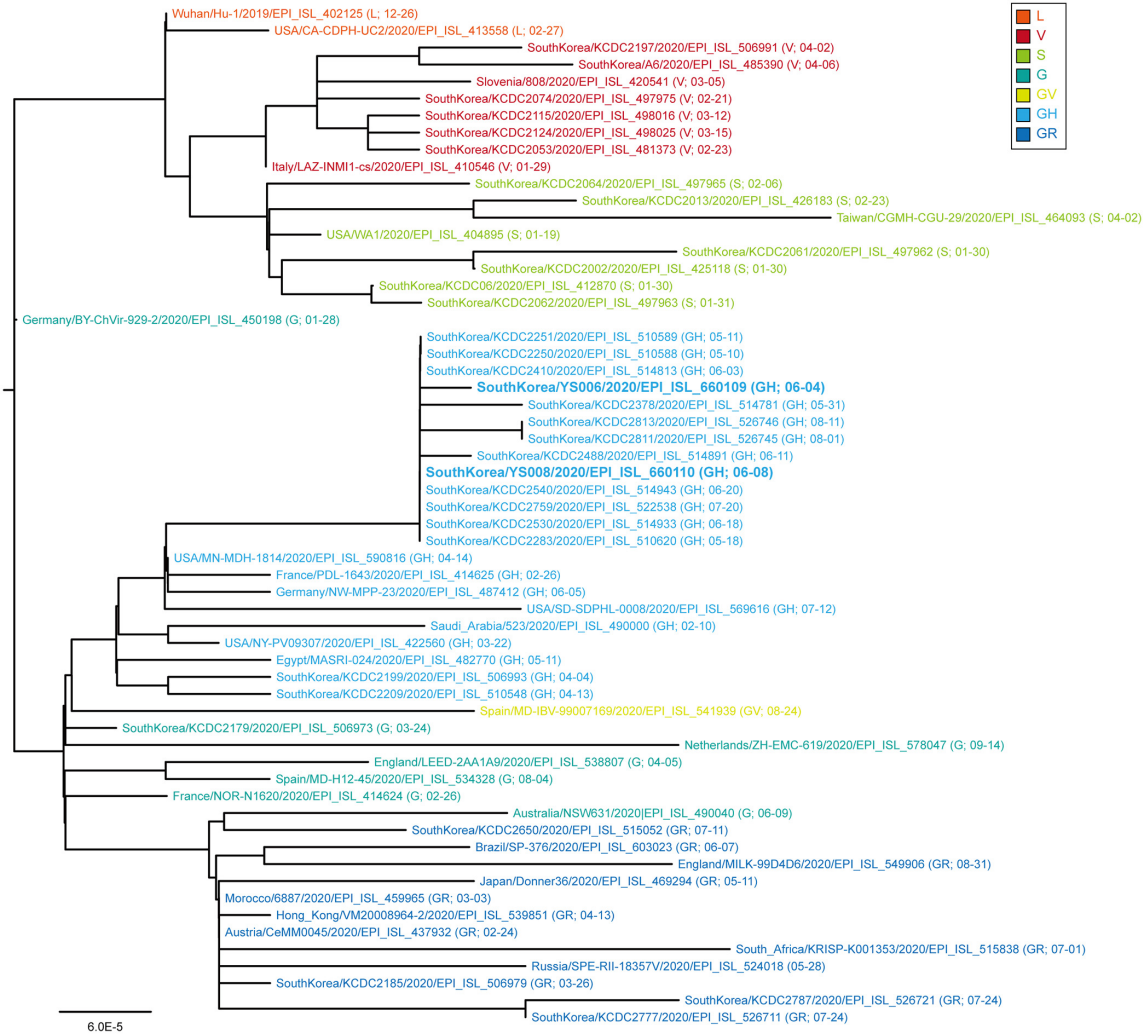
Phylogenetic tree of the genome sequences of SARS-CoV-2/human/KOR/YS006/2020 and SARS-CoV-2/human/KOR/YS008/2020, along with 58 other randomly chosen sequences, including 30 sequences of South Korean isolates of SARS-CoV-2 retrieved from Nextstrain (nextstrain.org). Multiple sequence alignments were conducted with MUSCLE v3.8.31 (12), and the phylogenetic tree was constructed using the Molecular Evolutionary Genetics Analysis (MEGA) v7.0 software by the neighbor-joining method with 1,000 bootstrap replicates (13). Shown in parentheses after the strain name and GISAID accession number are the clade to which it belongs and the collection date (month-day) for each strain. The sequences reported in this announcement are shown in bold. The SARS-CoV-2 isolates are presented in seven different colors for the different clades (shown in the key), classified according to GISAID classification. The scale bar represents the number of nucleotide substitutions per site.

Using the QIAamp viral RNA minikit (Qiagen, Hilden, Germany), RNA was extracted from the virus, which had been purified by passaging the swab samples three times on Vero cells (ATCC CCL-81) by the limiting dilution method (4). Viral cDNA synthesized using ProtoScript II reverse transcriptase (New England Biolabs, Ipswich, MA, USA) was amplified as described previously (5, 6), using in-house-designed primer sets and the Illumina platform-based BTSeq SARS-CoV-2 whole-genome sequencing (WGS) kit (Celemics, Seoul, South Korea) for multiplex amplicon sequencing on a MiSeq sequencer (150-bp paired-end mode; Illumina, San Diego, CA, USA). After dual-index filtering and adapter trimming using in-house scripts, reads (69,447 and 66,754 reads for isolates YS006 and YS008, respectively) were mapped to the reference sequence of Wuhan/Hu-1/2019 (GenBank accession number MN988668) (nucleotides 1 to 29870) (7) with BWA v0.7.17-r1188 (8), generating consensus genome sequences of strains SARS-CoV-2/human/KOR/YS006/2020 (29,825 nucleotides) and SARS-CoV-2/human/KOR/YS008/2020 (29,826 nucleotides) isolated from patients 6 and 8, respectively, with average coverage depths of 98.65× and 95.5×, respectively. The consensus sequences for YS006 (nucleotides 16 to 29840) and YS008 (nucleotides 16 to 29841) had no indels. The nearly complete genomes of these isolates, which lack 15 nucleotides and 29 or 30 nucleotides from their 5′ and 3′ ends [excluding the poly(A) tail], respectively (99.85% horizontal coverage), were 99.96% identical to the reference sequence. They both have a genomic GC content of 38%. Phylogenetic analysis revealed that these two isolates belong to the GH clade, which is currently most prevalent worldwide (9, 10), according to the GISAID classification (11) (Fig. 1). There were a total of 6 and 7 amino acid substitutions for YS006 and YS008, respectively, in comparison to the reference strain (Table 1). Both strains had C-to-T and G-to-T nucleotide changes in the 5′ untranslated region (UTR) and the 3′ UTR, respectively. A unique S6L substitution in the envelope (E) protein differentiated the YS008 strain from the YS006 and Wuhan/Hu-1/2019 strains.

**TABLE 1:** Nucleotide and amino acid changes in the YS006 and YS008 strains, in comparison to the reference strain

Nucleotide position	Nucleotide in strain (clade):			Gene name^*b*^	Amino acid change^*c*^
	Hu-1 (L)^*a*^	YS006 (GH)	YS008 (GH)		
241	C	T	T	5′ UTR	
1059	C	T	T	nsp2	T85I
3037	C	T	T	nsp3	
11916	C	T	T	nsp7	S25L
14408	C	T	T	nsp12	P323L
16650	C	T	T	nsp13	
20675	A	T	T	nsp16	Q6L
23403	A	G	G	Spike	D614G
25563	G	T	T	ORF3a	Q57H
26261	C	C	T	E	S6L^*d*^
29179	G	T	T	N	
29779	G	T	T	3′ UTR	

a Reference strain Wuhan/Hu-1/2019 (nucleotides 1 to 29870).

b ORF, open reading frame; nsp, nonstructural protein; E, envelope; N, nucleocapsid.

c Amino acid changes present in the YS006 and YS008 strains, in comparison to the reference strain.

d A nonsynonymous substitution present only in the YS008 strain.

### Data availability.

The sequences of SARS-CoV-2/human/KOR/YS006/2020 and SARS-CoV-2/human/KOR/YS008/2020 were deposited in the NCBI database (GenBank accession numbers MW345824 and MW345825, respectively) and in the GISAID database (https://www.gisaid.org) (accession numbers EPI_ISL_660109 and EPI_ISL_660110, respectively). The raw reads for the YS006 and YS008 strains were deposited in the NCBI Sequence Read Archive (SRA) database (accession numbers SRR13153716 and SRR13153715, respectively).
